# From Multidrug- to Extensively Drug-Resistant Tuberculosis: Upward Trends as Seen from a 15-Year Nationwide Study

**DOI:** 10.1371/journal.pone.0063128

**Published:** 2013-05-09

**Authors:** Karolien Stoffels, Caroline Allix-Béguec, Guido Groenen, Maryse Wanlin, Dirk Berkvens, Vanessa Mathys, Philip Supply, Maryse Fauville-Dufaux

**Affiliations:** 1 National Reference Centre of Tuberculosis and Mycobacteria, Communicable and Infectious Diseases, Scientific Institute of Public Health, Brussels, Belgium; 2 Genoscreen, Lille, France; 3 Belgian Lung and Tuberculosis Association, Brussels, Belgium; 4 Department of Biomedical Sciences, Institute of Tropical Medicine, Antwerp, Belgium; 5 INSERM, U1019, Lille, France; 6 CNRS UMR 8204, Lille, France; 7 Center for Infection and Immunity of Lille, Institut Pasteur de Lille, Lille, France; 8 Université Lille Nord de France, Lille, France; Hopital Raymond Poincare - Universite Versailles St. Quentin, France

## Abstract

**Background:**

Emergence of extensively drug-resistant tuberculosis (XDR-TB) represents an enormous challenge to Public Health globally.

**Methods:**

Progression towards XDR-TB was investigated in Belgium, a country with a typically low TB incidence, by analyzing the magnitude, characteristics, and treatment success of multidrug-resistant tuberculosis (MDR-TB) through a population-based study from 1994 to 2008.

**Results:**

Among the 174 MDR-TB patients, 81% were foreign-born, 48% of these being asylum seekers. Although the number of MDR-TB patients remained stable through the study period at around 15 new cases annually, frequencies of resistance of the patients’ first MDR-TB isolate to second-line drugs increased, as well as the total number of antibiotics it was resistant to (p<0.001). XDR-TB cases were detected from 2002 onwards. For 24 patients, additional resistance to several second-line drugs was acquired during treatment. Molecular-guided investigations indicated little to no contribution of in-country clonal spread or exogenous re-infection. The increase of pre-XDR and XDR cases could be attributed to rising proportions of patients from Asia and Central and Eastern Europe (p<0.001) and an increase in the isolation of Beijing strains in these groups (p<0.001). Despite augmented resistance, the treatment success rate improved from 63.0% to 75.8% (p = 0.080) after implementation in 2005 of improved surveillance measures and therapeutic access.

**Conclusions:**

Increasing severity in drug resistance patterns leading to more XDR- and “panresistant” TB cases in a country with a low TB incidence like Belgium represents a strong alert on worsening situations in other world regions and requires intense public health measures.

## Introduction

A major concern in the control of tuberculosis is the spread of multidrug-resistant tuberculosis (MDR-TB), caused by *Mycobacterium tuberculosis* resistant to at least isoniazid and rifampicin. A particularly dangerous form is the so-called XDR-TB (extensively drug resistant) [1], with additional resistance to any fluoroquinolone and to one of three injectable second-line drugs (amikacin, capreomycin, kanamycin) [2]. Recently, “panresistant” strains, resistant to all first- and second-line drugs with a proven activity against *M. tuberculosis*, have been reported [3]–[5]. Treatment failures observed with XDR-TB patients and the internationally increasing occurrence of “panresistant” strains [4], [6] risk to throw the fight against tuberculosis back to the pre-antibiotic era.

The latest estimates of the World Health Organisation (WHO) arrive at 650 000 prevalent MDR-TB cases among the 12 million tuberculosis cases worldwide [7] and 440 000 MDR-TB incident cases, of which 81 000 (18.4%) occurred in the WHO European region [8]. The increasing percentage of MDR-TB throughout Europe is alarming. Among these cases, Eastern Europe in particular contributes to the high proportion of XDR-TB [9].

A few longitudinal studies have reported the contribution to XDR-TB of additional resistance acquisition or transmission during treatment of MDR-TB, the upward trends over time of pre-XDR-TB in some MDR-TB patient populations, or the consequences of drug resistance severity on treatment outcomes of MDR-TB patients [10]–[13].

Here, we performed a retrospective study collectively looking at these parameters on the MDR-TB population over 15 years (sampling date between 1994 and 2008) in Belgium. This country has a TB situation representative of that of a number of other low TB incidence countries. In Belgium, the case notification rate decreased from 14.9 cases per 100 000 inhabitants in 1994 to an all-time low of 9.4/100 000 in 2008. Within this period the number of MDR-TB patients remained more or less stable [14] but the country is a migration crossroad, and immigration from Eastern and Central Europe is increasing. With such a context, our goal was to evaluate the evolution of MDR-TB and the efficiency of recently implemented measures for improving surveillance, treatment access and follow-up of MDR-TB patients.

## Methods

### Patients’ Information and Epidemiological Investigation

Demographic and clinical information was obtained from the National Tuberculosis Register, while information regarding patient management and treatment outcome was obtained through BELTA-TBnet, a project of the Belgian Lung and Tuberculosis Association (BELTA) set up by the federal government in 2005 to provide the best possible care to MDR-TB patients, ensuring free access to all necessary anti-TB treatment. All data accessed in the context of the present study had not been collected for research purposes but as part of the routine data collection for epidemiological surveillance, as stated in the Public Register dated 25/04/1997 in accordance with article 18 of the law of 08/12/1992 of the Belgian Government regarding the protection of the privacy of the individual when dealing with personal data. The aforementioned registration in accordance with the Belgian Privacy Commission stipulates in its §9 that no written informed consent from the patients is required for the collection and analysis of epidemiological data and treatment success.

Patients from Central and Eastern Europe were grouped for calculations.

### Clinical Isolates

Isolates were collected through the surveillance network of MDR-TB implemented in Belgium since 1994 and analyzed in the Belgian National Reference Centre of Tuberculosis and Mycobacteria (Scientific Institute of Public Health). For each of the 174 patients retained as true multidrug-resistant cases during the study period (1994–2008), only the first MDR isolate was included in the calculations of antibiotic resistance and cluster analysis.

### Drug Susceptibility Testing (DST)

DST was performed by the proportion method of Canetti [15] up to 2000 and thereafter in the BACTEC^TM^MGIT960 (Becton-Dickinson) for first-line drugs and in the radiometric BACTEC^TM^460 TB system [16] for second-line drugs like amikacin, ofloxacin, rifabutin, prothioamid, capreomycin and occasionally streptomycin, para-amino-salicylic acid, ciprofloxacin, clofazimin, clarithromycin and linezolid.

### Mutation Analysis

Mutations associated with resistance to rifampicin were identified by INNO-LiPA® RIF-TB (Innogenetics, Belgium) or by sequencing the 81 bp core region of the *rpoB* gene. Isoniazid resistance related mutations were determined by sequencing a 518 bp fragment of the *katG* gene and 204 bp of the *mabA-inhA* region, or by a multiplex PCR [17]. A 700 bp region of the *pncA* gene was investigated by sequencing for its association with pyrazinamide resistance [18], [19].

### DNA Fingerprinting and Cluster Analysis

Three different genotyping methods were performed: IS*6110*-RFLP [20] up to 2006, spoligotyping [21] and standardized 24-locus-based MIRU-VNTR typing [22]. Strain genetic groups were identified using the international spoligotyping database (SITVIT2) [23], and the MIRU-VNTR*plus* web application [24]. Cluster analysis was performed using BioNumerics (Applied Maths, Belgium).

A strain cluster was defined as two or more clinical isolates with identical RFLP, spoligotype and 24-locus MIRU-VNTR patterns, and identical mutations in the *rpoB*, *katG* and the promoter region of *inhA* genes. These mutations were considered as additional criteria for MDR-TB transmission, particularly for Beijing isolates with the same spoligotype and 24-locus MIRU-VNTR pattern.

### Statistical Analysis

Statistical analyses were carried out in Stata/MP 11.2 [25]. A generalized linear model was employed, using a logistic regression for prevalence data and a Poisson model for count data. Possible presence of extra-binomial model was verified and, if demonstrated, a negative binomial model was used. All models started fully saturated and the most parsimonious model is reported. A cut-off p-value of 0.05 was used throughout.

## Results

### Characteristics of the MDR-TB Population

During the 1994–2008 study period, a total of 174 patients (1.8% of all registered TB patients with a DST result) were retained as true MDR-TB cases, after exclusion of 19 persons considered to be false positive as the result of laboratory cross-contamination from high bacillary load smear positive pulmonary samples (11 occurrences each involving 1 to 5 samples in 7 different laboratories). It occurred particularly during the first years of the study and a revision of the clinical samples decontamination procedure was suggested to the concerned laboratories.

The 174 patients represent 80.6% of the culture positive MDR-TB patients reported in the Belgian TB register during the study period. For the remaining 19.4%, the culture was not sent to the Reference Laboratory or was too contaminated by other organisms to be further analysed.

Sixty-six percent of the patients were male, the mean age was 36 years (67.2% of the MDR-TB patients were in the 15–39 years age group) and 81% were foreign-born. Among the latter, 87.9% (123/140) came from highly endemic TB countries (defined as countries with an estimated prevalence of >100/100 000 inhabitants according to WHO [7]), and 47.9% (67/140) were asylum seekers. Thanks to a targeted TB screening of this last group, for 76.8% (43/56) of the asylum seekers with known date of arrival, MDR-TB diagnosis was established within 1 month, while it was made more than one year after arrival for 55.2% (16/29) of the other foreign-born. HIV test results were available for 66.1% of the patients: 16.5% tested were HIV positive.

Previous treatment history was known for 139 patients. Among the patients whose strain showed a pre-XDR or XDR resistance pattern, 80.0% (20/25) had been treated before, versus 44.7% (51/114) of the patients harbouring a strain resistant to first-line drugs only.

### Increase in Resistance Pattern of the Primary Isolate through the Study Period

**Figure 1:**
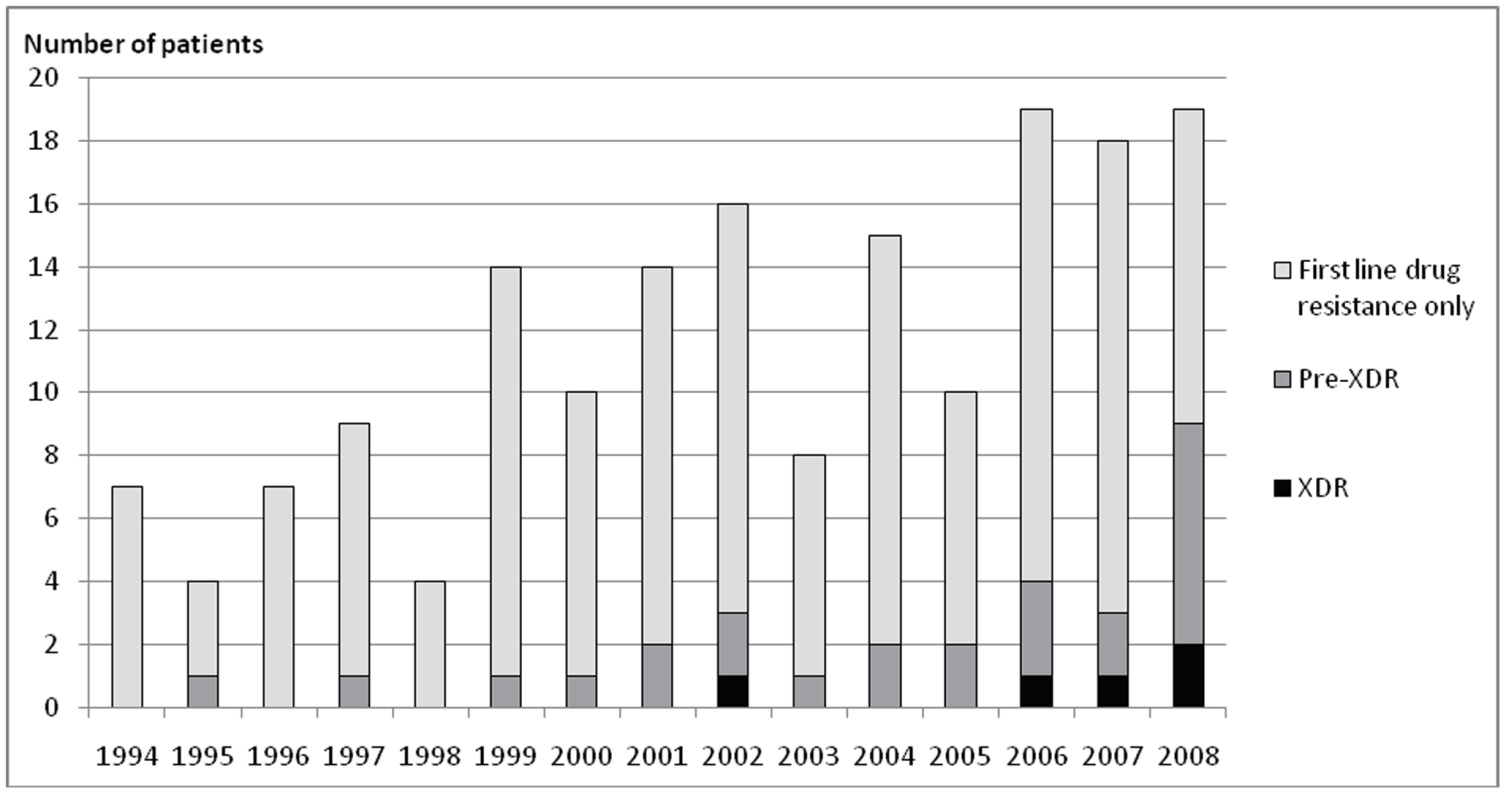
Number of MDR with first-line drug resistance only, pre-XDR and XDR isolates in the study cohort.

The first MDR-TB isolate obtained from each patient showed resistance to ethambutol, pyrazinamide, rifabutin, amikacin and ofloxacin in respectively 66.1%, 50.0%, 73.8%, 11.8% and 8.8% of the tested cases. An increase of resistance over time was observed for each of these drugs, and reached statistical significance (p<0.05) for ethambutol, amikacin and ofloxacin. Moreover, among the 170 MDR isolates for which at least ofloxacin and amikacin susceptibility results were available, the number of antibiotics each isolate was resistant to significantly increased over time (p<0.001). A total of 25/170 (14.7%) pre-XDR cases and 5/170 (2.9%) XDR cases were observed, and their proportion increased over time as well, to reach 36.8% for pre-XDR and 10.5% for XDR in 2008 (figure 1).

### Changes Over Time in the Phylogenetic Distribution of the Isolates and Drug Resistance Mutation Frequencies

Five genetic groups comprised 85.6% (149/174) of all clinical isolates: Beijing (28.2%), LAM (20.7%), Haarlem (10.3%), and generic Euro-American T1 (16.1%) and T2 (10.9%) groups. A statistically significant increase over time of Beijing profiles was observed (p<0.001), linked to their association with patients from Asia (15/24, p<0.001) and Central and Eastern Europe (30/60, p<0.001), the increase of MDR-TB patients from these two world regions (p = 0.009 and p = 0.038, respectively) and the increased proportions of Beijing strains isolated in these population groups (p = 0.064 and p = 0.051, respectively) through the study period.

Mutations associated with resistance to rifampicin were found in 97.1% (168/173) of the MDR-TB strains; 58.3% (98/168) of these mutations corresponded to a Ser531Leu change in the *rpoB* gene. This mutation was often found in Beijing isolates (p = 0.004) and in strains isolated from patients originating from Central and Eastern Europe (p = 0.007) but not from Asia (p = 0.502). The presence of the Ser531Leu mutation increased through the study period (p = 0.002) in the Beijing as well as in the non-Beijing isolates. It was also associated with resistance to rifabutin (p<0.001). From this study, the prediction that an isolate with this mutation is resistant to rifabutin is 95.0% (95% confidence interval: 88.0–98.0%). Concerning resistance to isoniazid, a mutation at nucleotide position 315 (S315T) of the *katG* gene or at position −15 (C-15T) in the promoter of the *inhA* gene, or at both locations were found in respectively 74.7% (127/170), 19.4% (33/170), and 7.1% (12/170) of the isolates. Presence of mutations in the *katG* gene also showed a significant increase over time (p<0.001).

In contrast, a much more diverse array of mutations with no specific time trend was found in *pncA* gene in 73.1% (57/78) of the pyrazinamid resistant isolates.

### Acquired Resistance during Treatment

**Figure 2:**
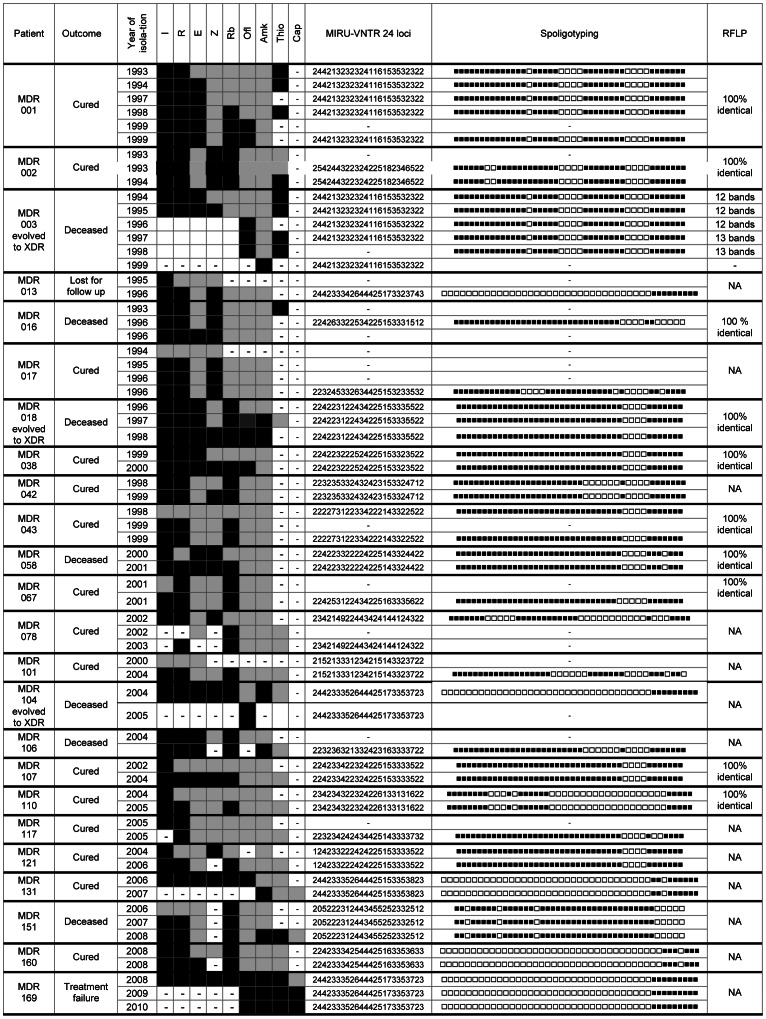
Evolution of the resistance profile of serial isolates obtained from 24 patients. For these 24 MDR-TB patients out of 37 with multiple isolates, resistance to additional drugs was observed during treatment. Legend: ▪ = Susceptible, ▪ = Resistant, −Test not performed, I: Isoniazid, R: Rifampicin, E: Ethambutol, Z: Pyrazinamid, Rb: Rifabutin, Ofl: Ofloxacin, Amk: Amikacin, Thio: Thioamide, Cap: Capreomycin NA = Not Applicable as less than 2 isolates were FLP profiled, so no conclusion possible.

Serial isolates were obtained from 37 patients including 4 patients whose first isolate was fully susceptible to first-line drugs (Figure 2). Twenty-four of the patients acquired additional resistance to 1 to 4 drugs (mean of 2 drugs) during treatment, over a period of 2 to 64 months (mean of 21 months). The isolates of 3 MDR patients from Italy (MDR003), Turkey (MDR018) and Russia (MDR104) evolved to an XDR pattern over a period of respectively 1, 2 and 5 years. All three patients died. The isolate of patient MDR169 from Chechnya, which was XDR upon arrival in Belgium in 2008, developed additional resistance in 2009 to become the first “panresistant” strain observed in Belgium. In addition to the drugs mentioned in Figure 2, the strain was resistant to ciprofloxacin, moxifloxacin, PAS, clarithromycin, clofazimin and linezolid.

In all cases, the genotypes of the successive isolates remained completely identical over the different typing methods tested (except for one patient where a single RFLP-band change was observed), indicating that additional resistance was not due to exogenous re-infection.

### Transmission of MDR-TB

**Figure 3:**
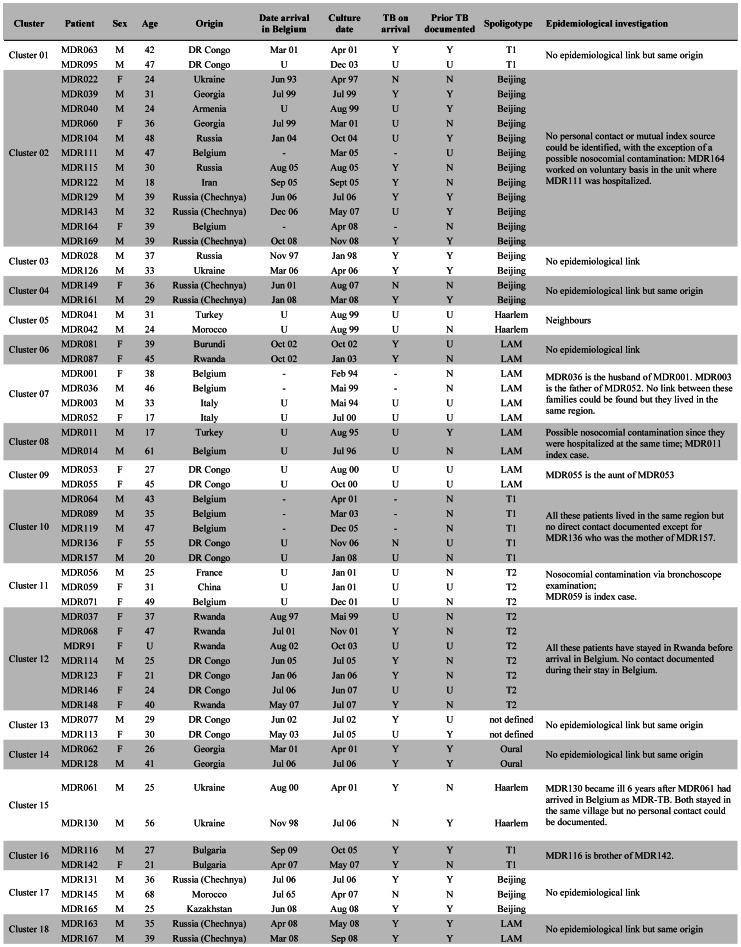
Characteristics of the 58 MDR-TB patients in strain-clusters and results of epidemiological investigation. Legend: Y = yes; N = no, U = unknown; − = not applicable.

Combining all genotyping techniques performed on the first MDR-TB isolate of the 174 studied patients, as well as interrogating mutations in the *rpoB*, *katG* genes and promoter region *inhA*, 134 different strain-genotypic profiles were identified: 116 patients (67%) were infected by a strain with a unique genotype while 58 patients, distributed in 18 clusters (Figure 3), shared their strain with at least one other patient. Out of these 58 patients, 48 were of foreign origin and 24 out of the 33 for whom this information was available had arrived in Belgium with MDR-TB already present.

The patients coming from Asia were less involved in clusters than the patients from the other regions (p = 0.019). However, no significant relation could be determined between the genetic lineage of the isolates and their clustering, *e.g.* the Beijing strains were not more clustered than strains of the other genetic families (p = 0.374).

An epidemiological link could be established for 6 clusters, while 3 partial links could be documented in 2 other clusters (Figure 3). These 9 links included 5 confirmed instances of intrafamilial transmission and one between neighbours, and 3 events of nosocomial infection (one strongly suspected between 2 hospitalized patients, one confirmed from a hospitalized patient to a hospital worker, and one contamination of 2 patients during bronchoscopy, described by Allix *et al*., 2004 [26]).

### Patients’ Outcome

**Figure 4:**
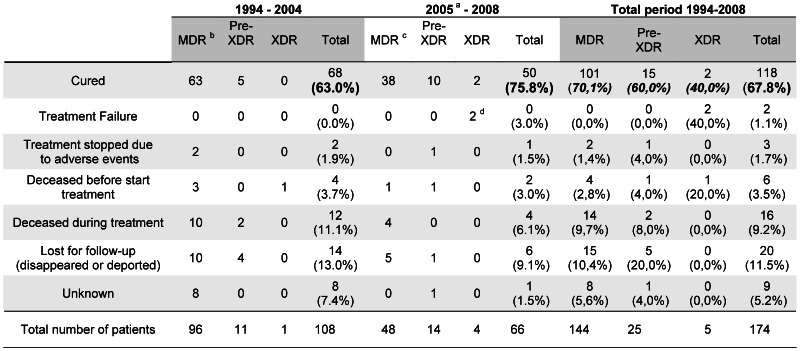
Treatment outcome of MDR (first-line drug resistance only), pre-XDR and XDR-TB patients. Legend: ^a^start of BELTA-TBnet in 2005 ^b^including 3 isolates with unknown amikacin susceptibility ^c^including 1 isolate with unknown amikacin susceptibility MDR: first-line drug resistance only Pre-XDR: MDR with additional resistance to either a fluoroquinolone or amikacin XDR: MDR with additional resistance to a fluoroquinolone and amikacin.

Treatment duration averaged 529 days, *i.e.* 17 months, based on the information available for 65 patients since 2004. The overall cure rate amounted to 67.8% (Figure 4) and varied according to the resistance of the isolate: 70% (101/144) for patients with first line resistance only, 60% for pre-XDR (15/25) and 40% (2/5) for XDR patients. A comparison of the cure rates of these 3 groups in the periods before (1994–2004) and after (2005–2008) the implementation of the BELTA-TBnet project showed that the treatment outcome improved substantially after the project start, both overall (from 63.0% to 75.8%; p = 0.080) and in each group separately but none of these differences reaches statistical significance.

**Figure 5:**
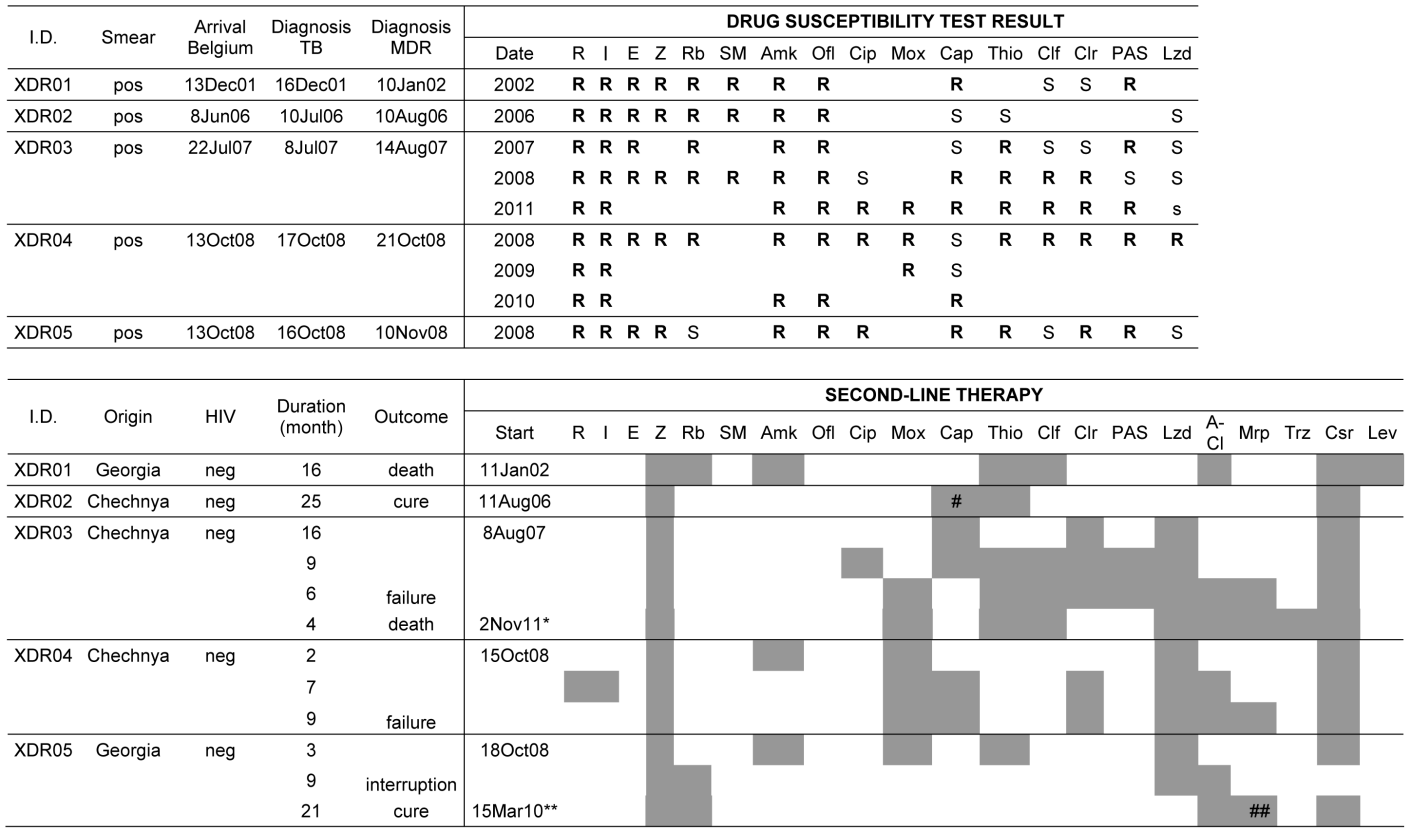
Patients with XDR-TB in their first isolate examined in Belgium, 1994–2008. Part I: patient characteristics and DST results. Part II: second-line therapy with treatment outcome. Legend: *New treatment regimen initiated after 19 months without anti-TB treatment **Retreatment after interruption of 5 months ^#^Stop after 8 months ^##^Stop after 7 months Antibiotics: R = Rifampicin, I = Isoniazid, E = Ethambutol, Z = Pyrazinamid, Rb = Rifabutin, SM = Streptomycin, Amk = Amikacin, Ofl = Ofloxacin, Cip = Ciprofloxacin, Mox = Moxifloxacin, Cap = Capreomycin, Thio = Thioamides, Clf = Clofazimin, Clr = Clarithromycin, PAS = para-aminosalycilic acid, Lzd = Linezolid, A-Cl = amoxicillin–clavulanate, Mrp = Meropenem, Trz = Thioridazine. The first treatment failure (XDR03) was simultaneously infected by two genetically different Beijing XDR strains, as detected by double alleles in 5 MIRU-VNTR loci. The second strain, not included in this study because not isolated from the patient’s first specimen, had additional resistance to capreomycin and clofazimine compared to the first one. After 31 months of unsuccessful hospital-supervised therapy, treatment was stopped. Nineteen months later, a new 9-drug regimen (including thioridazine) was prescribed but the patient died 4 months later. The second failure case (XDR04, the “panresistant” patient MDR169 mentioned in the text) returned to his home country after 18 months therapy, and subsequently died.

Three of the 5 patients whose first isolate collected in Belgium was already XDR presented an unsuccessful treatment outcome (Figure 5). One patient from Georgia died in 2003 after 16 months of second-line therapy and two patients, both from Chechnya, did not respond to long (31 and 18 months, respectively) very aggressive therapy with up to 10 anti-TB drugs that included linezolid and meropenem.

Two XDR patients cured. The first one, from Chechnya, responded well to a regimen of 25 months duration including pyrazinamid, capreomycin, ethionamide and cycloserin. The second one, from Georgia, had become bacteriologically negative after 3 months of second-line therapy but abandoned treatment after 12 months. Five months later, direct smear examination and culture were still negative but treatment was nevertheless restarted for an additional 21 months, with pyrazinamid, rifabutin, cycloserin, linezolid, meropenem and amoxicillin-clavulanic acid.

## Discussion

To our knowledge, this study is the first longitudinal nationwide survey of MDR and XDR-TB, covering a 15-year period (1994–2008), simultaneously integrating bacteriological and molecular data on the isolates, as well as epidemiological investigation, clinical aspects of the patients and treatment outcomes. The country studied has an epidemiological situation characteristic of that of many other low TB incidence countries [9]. In a context of an overall slowly decreasing TB incidence, the MDR-TB rates have been relatively stable over the last 15 years, at around 1.8% of the TB population tested for DST, but the resistance patterns of the isolates have become more severe. The study shows a majority of foreign-born patients (81%) and a significant increase over time both in resistance to ethambutol, amikacin and ofloxacin (p<0.05) and in the number of antibiotics the first isolates are resistant to (p<0.001), resulting in an upward trend of pre-XDR and XDR cases. Importantly, this trend has continued in the years after the end point of the present study. During the period 2009–2011, a total of 51 new MDR-TB strains were identified at first isolation: 36% presented a pre-XDR resistance pattern and 10 (20%) were XDR. Out of these 10, five were “panresistant”.

In-country transmission has played little to no role in fueling these trends, as indicated by the molecular-guided epidemiological investigations of the various patient strain clusters: 9 links only, in 8 clusters, detected or confirmed events of TB transmission. None of these involved XDR cases but one was a pre-XDR nosocomial transmission. In the other clusters, it was impossible to find any link between patients except that most cases in a same cluster were foreigners of the same geographic origin. The lack of proven links could reflect the difficulty of conducting retrospective field investigations in this specific population and/or the fact that many of these patients were already infected by a strain circulating in their country before they arrived in Belgium. The latter hypothesis is supported by data from the systematic screening of all asylum seekers for TB upon their arrival, and by the large diversity of strains with a unique genotype isolated in 67% of the MDR-TB patients.

Of note, no attempt has been made to systematically correlate the results of the cluster analysis to a possible classification of the cases as primary versus acquired resistance because the available information had, in most cases, been provided by the patients themselves without any documented proof.

Along the same lines, in contrast to what can be expected for high-TB incidence settings such as Uzbekistan [27], our genotyping results indicate that none of the 24 cases who acquired additional resistance to second-line drugs during treatment was due to an exogenous re-infection.

The increasing frequency of pre-XDR and XDR cases in Belgium appears to be driven mainly by the increased proportion of MDR-TB patients originating from Asia (p<0.001) and Central and Eastern Europe (p<0.001). Interestingly, this trend seems to be accompanied by a shift in the bacterial strain population structure in these patient groups, as seen by the increased proportions over time of the Beijing profiles isolated from these two world regions (p = 0.064 and p = 0.051, respectively). These findings are consistent with reports indicating both an increase in MDR- and XDR-TB rates, and an apparent selective expansion of some specific MDR- and pre-XDR-TB clones of *M. tuberculosis* Beijing in these world regions [28]. In accordance with the latter point, 22 (44.9%) of the 49 Beijing isolates identified in Belgium were caused by just two MIRU-VNTR genotypes: 12 by genotype 100–32, and 10 by genotype 94–32 (coded according to MIRU-VNTRPlus nomenclature, [29]).

Overall, a successful treatment outcome was obtained for 67.8% of the MDR-TB cases, above the 56.3% cure rate of MDR-TB patients observed in Europe in 2010 [9]. This rate in Belgium increased from 63.0% to 75.8% after the creation in 2005 of the BELTA-TBnet project, which more particularly ensured access to a number of antibiotics that have not been marketed as anti-tuberculosis drugs but may potentially prove to be useful additions to second-line treatment regimens, especially when treating pre-XDR and XDR cases, such as linezolid [30], meropenem-clavulanic acid [31], [32] and thioridazine [33], [34]. XDR-TB however remains difficult to treat. Only 2 patients were cured among the 5 cases diagnosed as XDR in their first specimen tested in Belgium. Nevertheless, this observation is in line with a systematic review of 13 studies [35] showing a successful treatment outcome in up to 65% of XDR-TB patients, particularly if not co-infected with HIV. In addition to BELTA-TBnet, other factors have contributed to the successful treatment outcome, such as the lower mean age of the MDR population but also the long hospitalization with controlled antibiotic administration and the special attention by qualified health care workers during the follow-up of these patients [36], in line with the national guidelines for the treatment of MDR-TB, updated in 2010 [37] according to the WHO recommendations [38].

In conclusion, while the annual MDR-TB case notification rate in Belgium has remained low, the severity of the drug resistance patterns has been increasing year by year, leading to more pre-XDR, XDR and “panresistant” TB strains. This trend mostly reflects the degrading situation of drug-resistant TB in Asia and Central and Eastern Europe, from which originate increasing numbers of MDR-TB patients in the country. Therapeutic outcomes have nevertheless been satisfactory and improved over the study period. This can be attributed to rapid diagnosis using early molecular detection of genotypic resistance, to molecular-guided cluster analysis confirming or disproving transmission and ensuring appropriate public health actions, as well as to enlarged therapeutic options and adapted structures for the patients’ long term treatment. In the face of the still growing proportions of pre-XDR, XDR and “panresistant” cases, these efforts must be maintained and intensified.
